# Polyamines and related signaling pathways in cancer

**DOI:** 10.1186/s12935-020-01545-9

**Published:** 2020-11-05

**Authors:** Jiajing Li, Yan Meng, Xiaolin Wu, Yuxin Sun

**Affiliations:** 1 Department of Otorhinolaryngology‐Head and Neck Surgery, China‐Japan Union Hospital, Jilin University, Changchun, Jilin Province China; 2 Department of Pathophysiology, Prostate Diseases Prevention and Treatment Research Center, College of Basic Medical Science, Jilin University, Changchun, China

**Keywords:** Polyamine, Cancer, Metabolism, Signaling pathway, Oncogene, ODC, SSAT, DFMO

## Abstract

Polyamines are aliphatic compounds with more than two amino groups that play various important roles in human cells. In cancer, polyamine metabolism dysfunction often occurs, and regulatory mechanisms of polyamine. This review summarizes the existing research on the metabolism and transport of polyamines to study the association of oncogenes and related signaling pathways with polyamines in tumor cells. Drugs that regulate enzymes have been developed for cancer treatment, and in the future, more attention should be paid to treatment strategies that simultaneously modulate polyamine metabolism and carcinogenic signaling pathways. In addition, the polyamine pathway is a potential target for cancer chemoprevention. As an irreversible suicide inhibitor of the ornithine decarboxylase (a vital enzyme of polyamine synthesis), Difluoro-methylornithine had been shown to have the chemoprevention effect on cancer. Therefore, we summarized and analyzed the chemoprophylaxis effect of the difluoromethylornithine in this systematic review.

## Background

**Fig. 1:**
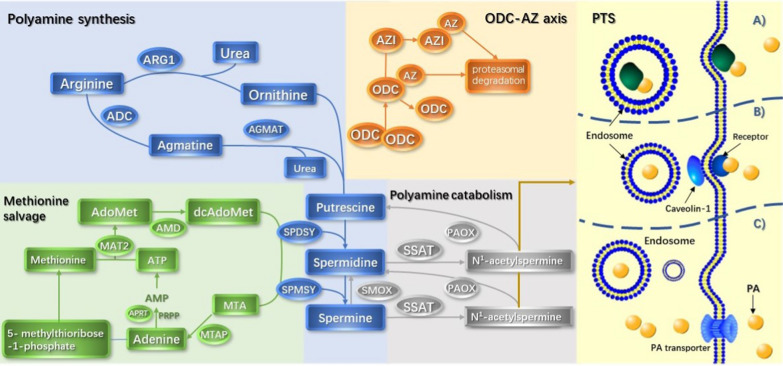
Polyamine biosynthesis and metabolic transport pathways. (1) Polyamine synthesis: arginine is converted into ornithine and agmatine, which is then catalyzed and decomposed into putrescine and urea by ornithine decarboxylase (ODC) and agmatine (AGMAT). Next, putrescine is converted to spermidine and spermine. (2) Methionine salvage: S-adenosylmethionine (dcAdoMet) decarboxylation provides aminopropyl for the formation of spermidine and spermine, and its product 5′-methylthioadenosine (MTA) is recovered to methionine through a series of enzymatic reactions. Subsequently, methionine is catalyzed by methionine adenosine transferase 2 (MAT2) and S-adenosylmethionine decarboxylase (AdoMetDC) to generate dcAdoMet. (3) ODC-AZ axis: the activity of ornithine decarboxylase is regulated by antizyme (AZ) and antizyme inhibitor (AZI). (4) Polyamine catabolism: spermidine and spermine are decomposed by spermidine/sperm-N-acetyltransferase (SSAT) to produce N-acetylspermidine and N-acetylspermine, respectively. (5) PTS (adapted from [18]): there are several different theories for the polyamine transport system: **a** Spermine combines with the heparan sulfate group in GPC1 on the cell surface and enters into the cell. **b** Polyamine transport is mediated by endocytosis and solute carrier transport mechanisms. **c** Polyamine is transported into the cell by a currently unknown transporter driven by membrane potential.

Polyamines are polycationic alkylamines commonly found in all living cells, of which the most common are putrescine, spermine, and spermine (in millimolar concentrations) [1, 2]. The flexibility in their charge distribution allows polyamines to combine with various negatively charged macromolecules, including DNA, RNA, proteins, and acidic phospholipids [3, 4]. Therefore, they play an important role in cell growth, proliferation, differentiation, migration, gene regulation, and the synthesis of proteins and nucleic acids, in addition to maintaining chromatin structure, regulating ion channels, maintaining membrane stability, and scavenging free radicals [5–7]. It has been shown that increased intracellular polyamine concentrations are associated with cell proliferation and tumorigenesis [8–14]. Polyamine metabolism is often dysregulated in cancers. In addition, the polyamine pathway is a downstream target for many oncogenes [15–17]. In normal physiological conditions, polyamines are regulated by a complex network of biosynthesis, catabolism, and transport systems (Fig. 1).

## Polyamine synthesis and metabolism

### Polyamine biosynthesis

Excess nitrogen and ammonia produced by protein breakdown or nitrogen compound synthesis in vivo can be eliminated by the urea cycle. During this process, arginine is catalyzed by arginase to produce ornithine, the substrate for the synthesis of urea and polyamines. The main pathway of polyamine biosynthesis is the decarboxylation of ornithine catalyzed by ornithine decarboxylase (ODC) to generate putrescine. After that, spermidine and spermine are produced by the enzymatic transfer reaction of spermidine synthetase (encoded by *SRM*) and spermidine synthetase (encoded by *SMS*). The decarboxylation of s-adenosine methionine (dcAdoMet) catalyzed by S-adenosine methionine decarboxylase (AdoMetDC; encoded by *AMD1*) provides the aminopropyl donor for the above reactions [19–21]. 5′-methyl thio-adenosine (MTA) is produced during spermine and spermine synthesis following the loss of dcAdoMet aminopropyl, which requires methionine recovery to methionine. MTA is converted to adenine and 5-methylthioribose-1-phosphate by 5′-methylthioadenosine phosphorylase (MTAP). Methionine is extracted from 5-methylthioribose-1-phosphate to form a substrate that binds to ATP. It then interacts with methionine adenosyltransferase 2 (MAT2) to form S-adenosylmethionine (AdoMet). S-adenosylmethionine provides aminopropyl for the production of spermidine and spermine [1, 22]. Recently, it was shown that arginine could be decarboxylated to produce agmatine in mammals, generating urea and putrescine under the action of agmatinase (AGMAT) [23].

Ornithine decarboxylase, a pyridoxal phosphate-dependent enzyme, is the rate-limiting enzyme for polyamine synthesis. The activity of ODC in cancer cells is reported to be consistently increased, demonstrating its close relationship to the occurrence and development of tumors. ODC protein levels are regulated by a variety of stimuli, including hormones, growth factors, oncogenes, and free polyamines [19]. Anti enzyme (AZ) combines with ODC monomers to prevent it from forming an active homodimer, thereby promoting its ubiquitin-dependent degradation by the 26S proteasome. AZ synthesis is influenced by antizyme inhibitor (AZI), a protein encoded by the *AZIN1* gene. The structure of this protein is similar to ODC, but it binds AZ more closely than ODC, thereby blocking the ability of AZ to inhibit ODC [24, 25].

### Polyamine catabolism

The level of polyamine is also regulated by its catabolism, which can prevent the excessive levels of polyamine in cells [26]. Spermidine/spermine-N-acetyltransferase (SSAT) respectively acetylates spermidine and spermine to produce N-acetylspermidine and N-acetylspermine [27]. These acetylated polyamines can form putrescine via oxidative deamination reactions catalyzed by polyamine oxidase (PAOX). Spermine oxidase (SMOX) catalyzes the oxidation of spermine to spermidine [1]. SSAT regulates the cellular polyamine content. SSAT is highly regulated according to changes in the polyamine content to maintain the steady state of polyamines. SSAT levels are usually very low but easily enhanced by increasing free polyamines. SSAT activity can also be induced by a variety of other stimuli, including toxins, hormones, cytokines, non-steroidal anti-inflammatory agents, natural products and stress, and ischemia–reperfusion injury [27]. SMOX and PAOX are likely to produce large amounts of reactive oxides (ROS), leading to oxidative damage [28].

## Polyamine transport system

Polyamines are protonated at a physiological pH, which hinders their passive transport through the plasma membrane. Instead, the active transportation of polyamines through the polyamine transport system has the characteristics of energy and temperature dependence and saturation at low concentrations [22]. At present, there are several theories regarding polyamine transport.

One theory suggests that heparan sulfate and glypican 1 (GPC1) coordinately transport spermine. Spermine interacts with the heparan sulfate group in GPC1 on the cell surface and enters into the cell. Spermine is then released through NO-mediated oxidation to act on cells [29].

Another view is that polyamine transport is mediated by endocytosis and solute carrier transport mechanisms. Polyamines bind to polyamine binding proteins and are internalized by endocytosis. Caveolin-1 knockout promotes endocytosis and increases the frequency or amount of polyamine internalization but does not change the affinity of the polyamine to bind to the cell surface [30]. According to this theory, SLC3A2 exports putrescine and acetylated polyamines via diamine/arginine exchange activity [31]. In the case of high extracellular putrescine and low intracellular putrescine, the concentration gradient can drive SLC3A2 to become a carrier of putrescine [30]. Subsequently, it was discovered that caveolin-1 negatively regulates polyamine uptake by inhibiting GST∏ secretion by stimulating actin remodeling and endocytosis [32].

Alternatively, it is thought that polyamines are transported into cells through an unrecognized transporter driven by membrane potential. Polyamine penetrates the cell through the plasma membrane. The accumulated polyamines are subsequently localized in polyamine isolation vesicles, which relies on the vacuolar ATPase pH gradient and proton exchange, illustrating the two-step mechanism of polyamine transport and vesicle chelation [33].

Research on polyamine transporters has been progressing. There is evidence that Membrane transporters may mediate the transport of polyamines. For example, the transport proteins encoded by *SLC22A1, SLC3A2, SLC22A16, and SLC12A8A* can transfer putrescine or spermidine and spermine into cells. *SLC18B1* is a recently identified gene in the family of vesicle amine transporters. This protein is responsible for the storage and release of polyamine vesicles and functions as a vesicle polyamine transporter [34].

Recent studies have shown that deficiency in ATP13A2, a late endolysosomal transport protein, can interfere with lysosomal polyamine transfer. ATP13A2 promotes the uptake of polyamines by cells through endocytosis and transports them to the cytoplasm, highlighting the role of endolysosomes in the uptake of polyamines into cells [35].

## Polyamines and cancers

As mentioned earlier, polyamine dysregulation has been found in a variety of cancers. For example, polyamine metabolism plays a key role in cell death and proliferation in breast cancer. And there is evidence that polyamines facilitate the interactions of transcription factors, such as estrogen receptors and nuclear factor kB, with their specific response element and are also involved in the proliferation of ER-negative and highly invasive models of tumor cells [11, 36]. Polyamines are polycationic compounds that play a key role in almost all the steps of colorectal tumorigenesis. In the tissue of colorectal cancer, the polyamine content as well as the activities of two important enzymes in their biosynthesis such as ornithine decarboxylase and S-adenosylmethionine decarboxylase, are increased 3 ~ fourfold over that found in the equivalent normal colonic tissue. The researchers found that polyamines could be a target for chemoprevention. Therefore, it can be deduced that influencing polyamine metabolism by drugs and diet is able to reduce cancer risk [14, 37, 38]. The polyamine content in prostate cancer was significantly higher than that in benign prostatic hyperplasia. Prostate cancer cells maintain the secretion of polyamines while proliferating, so they need a high level of polyamine metabolic flux. Polyamine metabolic pathway may be a target of prostate cancers [12, 38]. Some researchers have proved that polyamines play an important role in the early promotion stage of skin tumor by building transgenic mouse model. Polyamines can stimulate epidermal proliferation, change the differentiation of keratinocytes, increase neovascularization, and increase the synthesis of proteins in extracellular matrix in a manner similar to wound healing. The increase of polyamine level can not only activate epidermal cells, but also subcutaneous stromal cells, thus promoting the development and progression of skin tumors. Targeted ornithine decarboxylase has been shown to prevent non-melanoma skin cancer in human [10, 13].

Therefore, polyamines and their metabolites are often regarded as cancer biomarkers. In recent years, metabonomics technology can more sensitively observe the changes of polyamine synthesis and metabolism in cancer. Polyamines and their metabolites in urine and plasma can be used as biomarkers of occurrence and progression in a variety of tumors, such as breast cancer, lung cancer, colorectal cancer, ovarian cancer, prostate cancer and pancreatic cancer [22]. In addition, it has been observed that polyamines improve the malignancy of tumors and the invasion and metastasis of cancer cells, and reduce the anti-tumor immune function of immune cells [39, 40].

## Polyamine-associated oncogenes and related signaling pathways

The mechanism by which polyamines affect the occurrence and development of cancer has been the focus of many researchers. This review summarizes the oncogenes that interact with polyamine pathway, as shown in Table 1.

**Table 1:** Interaction between polyamines and oncogenes

Oncogene	Activated	Suppressed	Effect of PAs	PAs feedback regulation
MYC	Polyamine biosynthase genes(*ODC1, AMD1, AZIN1, DHPS, EIF5A, MAT1B, SMS, SMOX, SRM*); Polyamine transporters SLC3A2; eIF5A; arginase	Polyamine catabolism enzyme genes (*OAZ1,OAZ2, OAZ3, PAOX* and *SAT1*)	Activation	Polyamines promote the translation and expression of MYC
P53	SSAT	Urea cycle enzyme genes (*CPS1, OTC* and *ARG1*); ODC	Suppression	Polyamine depletion could increase the expression of p53.Spermine may activate p53 transcription by inducing autophagy
RAS	ODC	Caveolin-1, SSAT	Activation	
RAF	Polyamine transport system			Polyamines can change the phosphorylation of RAF through casein kinase 2 (CK2), thus acting as inhibitors (spermine) or activators (spermidine or putrescine combined with spermine) of Raf
MEK				Cellular polyamines regulate the expression of MEK-1 at the post transcriptional level through the RNA binding protein HuR in IECs
AKT				The ODC inhibition and SSAT expression can block the activity of AKT/GSK3-β/β-catenin pathway
MTOR	MTORC1 can maintain the stability of ODC mRNA and increase the activity of AdoMetDC	In the absence of amino acids, the activity of mTORC2 is necessary for the synthesis of AZ	Activation	In the absence of amino acids, polyamines increase mTORC1 and mTORC2 activity
Rac/RhoA				Polyamine depletion leads to localization of Rac1 and RhoA in the nucleus and perinuclear region, which reduces their activity
JUN/FOS	ODC、MAT2		Activation	
LIN28				Polyamines can regulate LIN28 via the tyrosine-modified eIF-5A

### MYC

The *MYC* transcription factor family is one of the central and most studied groups in cancer. In 1992, the Cleveland team first reported that c-myc could regulate ODC expression at the transcription level [41]. The *ODC1* gene contains a *MYC* binding site in its promoter, which contains a conserved E-box motif. A single nucleotide polymorphism in the E-box region of the *ODC1* gene affects the binding of *MYC* and *MAD* to *ODC1* and is related to the recurrence of colon cancer [42].

There are frequent mutations of one or more *MYC* genes in various cancers, and the overexpression of *ODC1* is regulated by *MYC* activation. For example, in neuroblastoma, bioinformatics analysis of a large number of human neuroblastoma samples showed that genes associated with polyamine biosynthesis, including *ODC1, AMD1, ARG1, AZIN1, DHPS, EIF5A, MAT1B, SMS, SMOX*, and *SRM*, were upregulated in *MYCN*-amplified/upregulated neuroblastoma. In contrast, *OAZ2, PAOX*, and *SAT1* genes involved in polyamine catabolism were downregulated in tumors. *MYCN* also regulated the key polyamine transport protein SLC3A2, and their expression levels were positively correlated [43–46]. A recent study had demonstrated that downregulation of both SMS and *MYC* synergistically induces apoptin Bim expression in colorectal cancer cells, indicating that combined inhibition of SMS and *MYC* signaling may be an effective therapy for colorectal cancer [47].

Other studies found that polyamine negative feedback regulates the expression of *MYC*. For example, it has been demonstrated that putrescine triggers the transcription of c-myc mRNA in renal cells of Kirsten sarcoma virus-infected rats, while difluoromethylornithine (DFMO) inhibits ODC activity and blocks the transcription of c-myc [48]. Polyamines in rat intestinal epithelial cells enhance the association between HuR and the 3′-untranslated region of c-myc mRNA by increasing the HuR-mediated phosphorylation of CHK2, thereby promoting c-myc translation [49]. In addition, polyamines drive the expression of c-myc by inducing the four-chain structure of c-myc to form a transcriptionally active motif with unique molecular recognition properties [50].

### p53

The combination of p53 with CPS, OTC, and ARG, the key enzymes of the urea cycle, downregulates the transcription of these enzymes. It inhibits urea production and ammonia elimination in vitro and in vivo, thereby inhibiting tumor growth. In contrast, the downregulation of these genes activates p53 through a mechanism mediated by MDM2.The accumulation of ammonia leads to a significant decrease in the mRNA translation of the polyamine biosynthesis rate-limiting enzyme ODC, which inhibits polyamine biosynthesis and cell proliferation. However, ammonia does not affect the ubiquitination state of ODC proteins or the interaction between ODC monomers. Similarly, p53 deletion increases the overall level of ODC monomers and dimers, and thus p53 regulates ammonia metabolism through the urea cycle to control polyamine biosynthesis [51].

There are two p53 binding sites in the promoter region of *SAT1*, indicating that the *SAT1* gene is a transcriptional target of p53. P53 mediates the activation of *SAT1* expression, which induces lipid peroxidation and causes iron death under ROS-induced stress [52].

It has been found that spermine can induce autophagy, which is related to the activation of p53 transcription [53]. AdoMetDC is an essential enzyme for polyamine biosynthesis. Its inhibitor SAM486A leads to the rapid accumulation of the pro-apoptotic proteins p53 and MDM2 [54]. The inhibition of ODC increases the phosphorylation of p53 and MDM2, and the resistance to apoptosis [55]. Polyamines play an important role in maintaining the integrity of the normal intestinal epithelium. The depletion of polyamines can significantly enhance the cytoplasmic abundance of HuR, which specifically binds to the untranslated region of p53 mRNA. Therefore, polyamines can control the stability of p53 mRNA and affect the level of p53 protein [56].

### RAS/RAF/MEK pathway

#### RAS

The transcription and translation of ODC is controlled by RAF/MEK/ERK pathways [57]. Ras activation enhances IRES-mediated ODC translation activity independent of cap. This regulation is dependent on the phosphorylation state of eIF4E. Dephosphorylation of eIF4E by inhibition of MEK or MNK1/2 inhibitor the activity of ODC-IRES [58].

The activated *K-RAS* significantly increases the uptake of polyamines by colon cancer cells. Activated *K-RAS* changes the subcellular distribution of the uPAR ligand uPA, which activates Src. Activation of Src increases the phosphorylation of caveolin-1, which is a negative regulator of caveolin-1 endocytosis [59].

It has been shown that *K-RAS* mutations can inhibit SSAT expression through the peroxisome proliferator activated receptor-γ (PPARγ) response element, which is located at + 48 bp relative to the transcription start site of the SSAT, to maintain a high level of polyamine in transformed cells [17].

In *H-RAS* transformed cells, the regulation of multiple cytokines could affect the expression of key enzymes in polyamine anabolism. For example, ODC is regulated by basic fibroblast growth factor (bFGF), transforming growth factor β (TGF-β), platelet-derived growth factor (PDGF), and cAMP [60–63]. Similarly, S-adenosylmethionine decarboxylase expression is regulated by epidermal growth factor (EGF) and bFGF [64].

#### RAF

Missense mutations in the *BRAF* oncogene occur in more than 50% of malignant melanomas [65]. *BRAF* inhibitors against *BRAF*-mutated tumors are susceptible to drug resistance [66]. An in vitro study demonstrated that compared with wild-type *BRAF* melanoma cells, mutant *BRAF* melanoma cells showed stronger PTS activity and were more sensitive to PTS-targeted cytotoxic drugs [67].

Polyamines can change the phosphorylation of Raf through casein kinase 2, thus acting as inhibitors (spermine) or activators (spermidine or putrescine combined with spermine) of Raf [5]. Some experiments indicate that under the condition of satisfying the selective susceptibility, an increase in ODC results in an increase in polyamines, which in combination with the activation of the RAF/MAPK pathway, can transform normal keratinocytes into invasive malignant cells [68].

#### MEK

MEK-1 is a key effector in HuR-induced anti-apoptotic programs in intestinal epithelial cells (IECs). Cellular polyamines regulate the expression of MEK-1 at the post-transcriptional level through the RNA binding protein HuR in IECs. MEK-1 mRNA can be stabilized by inhibiting the decrease in cell polyamine levels caused by ODC, and its translation can be promoted by enhancing the interaction between HuR and the 3′-untranslated region of MEK-1 mRNA [69].

### AKT

AKT is involved in several cell processes, such as cell survival, growth, and migration. It is known that the inhibition of ODC expression can block the activation of the AKT pathway in acidosis microenvironments. ODC is co-expressed with β-catenin in liver cancer. The expression and nuclear localization of β-catenin decreases after ODC inhibition. Blocking the metabolism of polyamines by treatment with polyamine conjugates inhibits the activity of AKT and apoptosis-related proteins [70].

In addition, high levels of polyamines activate AKT. According to previous reports, exogenous polyamines can induce cancer cell proliferation and migration through AKT-mediated pathways [71]. Polyamines regulate hypoxia-induced apoptosis of endothelial cells through the PI3K/AKT pathway, which is of great significance to the regulation of hypoxia driven neovascularization [72]. It has also been shown in several human hepatocarcinoma and colon cancer cell models that SSAT expression mediated polyamine depletion can significantly inhibit the expression of p-Akt, p-GSK3β, and β-catenin nuclear translocation, thus inhibiting the growth, migration, and invasion of cancer cells [73].

### mTOR

mTOR forms two different complexes named mTOR complex 1 (mTORC1) and complex 2 (mTORC2). Polyamines are necessary for the synthesis of AZ. In the absence of amino acids, the activity of mTORC2 is necessary for the synthesis of AZ. Because mTORC1 is inhibited, and mTORC2 is activated, the synthesis of total protein is inhibited, and the synthesis of AZ1 is increased through a cap-independent mechanism. In addition, it was subsequently demonstrated that putrescine, spermidine, and spermine all increased mTORC2 activity, whereas spermidine and spermine increased mTORC1 activity [74, 75]. mTORC1 inhibition reduces the association of the mRNA binding protein HuR with ODC transcripts, thereby destabilizing ODC mRNA [76]. In glioma cells, the activation of polyamine catabolism alters the location of mTOR, negatively affecting mTOR-mediated protein synthesis and leading to apoptosis [77]. It has been shown that spermidine can reduce apoptosis by promoting AMPK/mTOR-mediated autophagy flux [78].

Using comprehensive metabolomics methods in mouse and human prostate tumors, it was found that the protein level of AdoMetDC is increased in PTEN-deficient prostate cancer cells. PTEN is a tumor suppressor that is frequently mutated or lost in prostate cancer. Loss of PTEN function causes an abnormal response to growth factor (GF) stimulation through the PI3K signaling pathway, thereby activating mTORC1. mTORC1 inhibitors induce AdoMetDC downregulation, and at the same time, dcSAM production and polyamine synthesis are reduced. Mechanistically, activated mTORC1 indirectly blocks the proteasome degradation of pro-AdoMetDC and leads to phosphorylation at the S298 site, further stabilizing it. Then, the proenzyme self-processes into an active holoenzyme containing pyruvate, promoting an increase in the production of polyamines necessary for neoplastic growth [79].

### Others

#### Rac and RhoA

Studies have shown that inhibition of RhoA activity and depletion of polyamines inhibit cell migration, causing changes in the actin cytoskeleton. Polyamine depletion leads to Rac1 and RhoA localization in the nucleus and perinuclear region, which reduces the levels of Rac1 and RhoA protein in the cytoplasm and at the plasma membrane, significantly reducing their activity. These findings provide novel insights into the mechanisms by which ODC and polyamines regulate cytoskeletal dynamics during cell proliferation and transformation [80–82].

Polyamines increase intracellular free Ca^2+^ concentrations by controlling voltage-gated K + channel expression and membrane potential (E (m)) during intestinal epithelial repair. This increases the binding of GTP to RhoA, which can interact with and activate Rho kinase during intestinal epithelial repair [83].

#### JUN and FOS

It has been reported that *Helicobacter pylori* activate polyamine-dependent mechanisms through specific MAPK pathways to induce macrophage apoptosis. *H. pylori* activate ERK, and the translocation of p-ERK to the nucleus can lead to activation of activator protein 1 (AP-1). AP-1 consists of a phosphorylated c-Fos/c-Jun heterodimer that binds to the c-myc promoter in macrophages, thereby inducing the expression of c-myc and ODC and increasing polyamine production. Subsequently, the oxidation of spermine by spermine oxidase to produce hydrogen peroxide causes mitochondrial membrane polarization, which eventually leads to cell apoptosis [84, 85]. Similarly, in colon cancer, the expression of ODC, MAT2, FOS, and JUN in tumor tissues is higher than that in adjacent normal mucosa to provide polyamines for tumor cell proliferation [86].

#### LIN28/let-7 pathway

The Lin28/let-7 pathway is involved in the metabolism of polyamines and plays a key role in the regulation of normal and cancer stem cell self-renewal. Lin28 and Wnt signaling pathways cooperate to promote the development of invasive intestinal and colon cancers [87]. The let-7 family is negatively regulated by the pluripotent factor Lin28. Polyamines can regulate LIN28 via the tyrosine-modified eukaryotic translation initiation factor 5A (eIF-5A), which uses spermidine as substrate, thus affecting specific aspects of tumorigenesis [88].

#### Hedgehog pathway

In Hedgehog pathway, signal transmission is controlled by patched (PTCH) and smoothed (SMO) receptors on the target cell membrane. At the post-receptor level, cytoplasmic regulators suppressor of fused (SUFU) and glioma-associated oncogene transcription factors are key mediators of the Hedgehog transcription [89]. In medulloblastoma, tumors in the sonic hedgehog subgroup show abnormal activation of Hedgehog signaling. SMO activation triggers the non-classical hedgehog signaling pathway associated with the energy sensor AMPK. Cell nucleic acid-binding protein (CNBP), a type of RNA binding protein, is the key factor for this reaction. Through phosphorylation modifications, CNBP increases its stability and close interactions with SUFU. The SUFU-CNBP complex binds to the 5′ untranslated region of ODC mRNA and promotes its translation, thereby increasing polyamine metabolism [90].

## DFMO in polyamine chemoprevention

Inhibition of ODC activity and polyamine synthesis is theoretically beneficial to cancer prevention. Based on preclinical and early clinical studies, DFMO is expected to be a promising chemical prophylactic. Some clinical trials have studied the chemopreventive effect of the polyamine inhibitor DFMO on cancer.

**Fig. 2:**
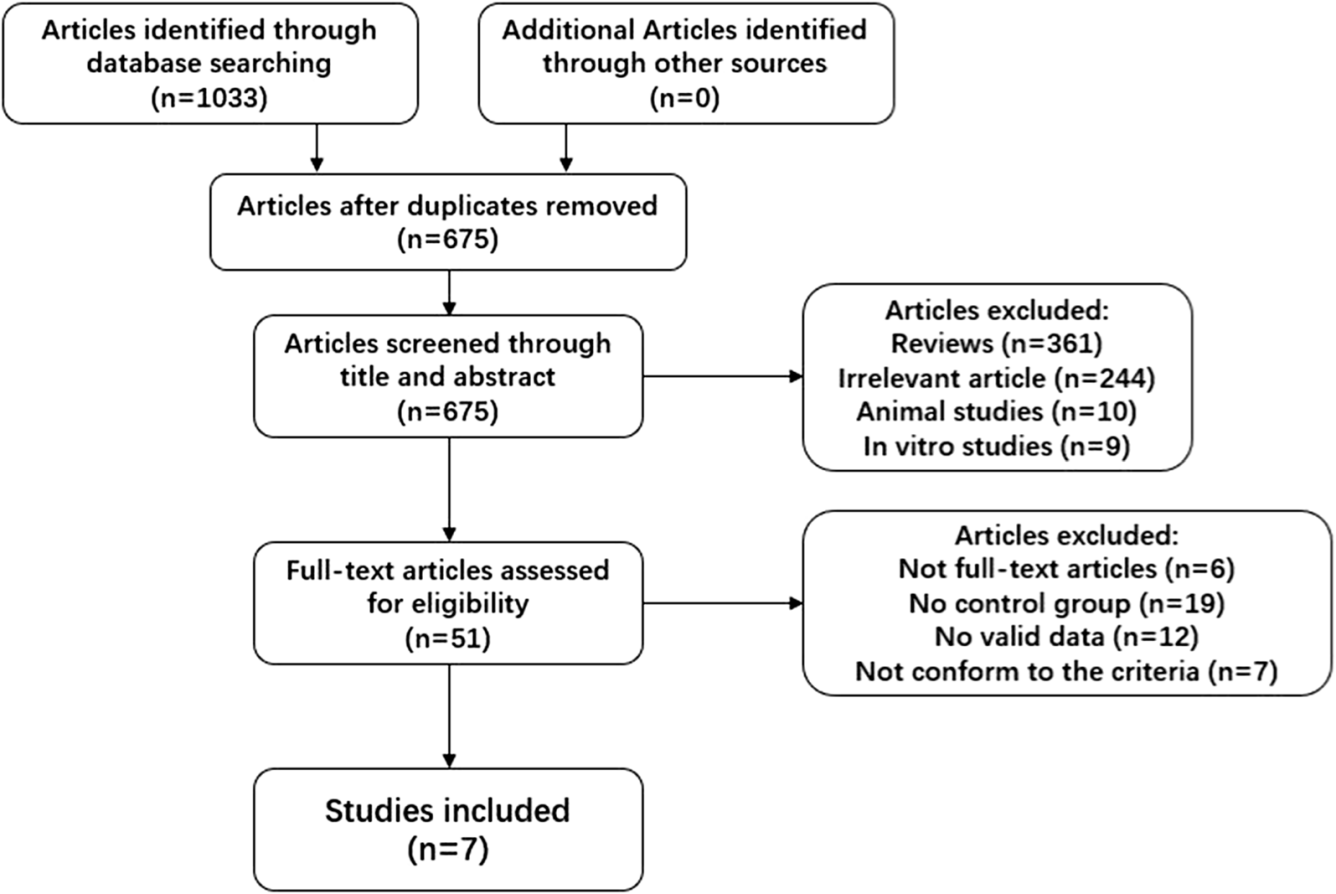
Flow chart of literature selection

This review used keywords to search the literature from several English databases (Pubmed, Embase, Cochrane library, Scopus, Web of Science) and selected the collected literature through the following inclusion criteria: (1) clinical randomized controlled trials, (2) studies set up groups that used and did not use DFMO, (3) the participants in the trial were treated cancer patients or high-risk groups prone to cancer, and (4) observation indicators related to the outcome may indicate remission or deterioration of the patient's condition. The literature screening process is shown in Fig. 2. In this process, two researchers screened the literature back-to-back at the same time, and after two checks, the results were confirmed to be consistent, the following documents were finally included. The details of the final included literature are shown in Table 2.

**Table 2:** **Information about included clinical trials**

	Disease	Population	Population characteristics		Observation target	Groups	Dosage	Duration	Outcomes
			Age	Gender (M/F)					
Lewis 2020 [90]	Neuroblastoma	Patients with HRNB who completed standard upfront therapy without progression were eligible for enrollment	Mean = 4.0	96 (61.1%)/60 (38.2%)	Event‐free survival (EFS) and overall survival (OS)	DFMO/No treatment	750±250 mg/m^2^/dose, twice daily	5 years	2 year EFS: DFMO:86.4% [95% CI, 79.3–94.2%]; No treatment:78.3% [95% CI, 69.5–88.3%]. 5 year EFS: DFMO: 85.2% [95% CI, 77.8–93.3%]; No treatment:65.6% [95% CI, 55.5–77.7%]. 2 year OS: DFMO:8.8% [95% CI, 96.4–100%]; No treatement:94.4%[95% CI, 89.3–99.9%. 5 year OS: DFMO:95.1% [95% CI, 90.5–99.9%]; No treatment:81.6%[95% CI, 73.0–91.2%]
Lynch 2016 [93]	Familial adenomatous polyposis	Study population was eligible for FAP diagnosis, requiring an evaluable colon and/or rectal segment and five or more colon polyps at baseline	Mean = 38 (18–63)	60 (54%) /52 (46%)	Average percentage change in adenoma polyp counts; Average percentage change in adenoma polyp burden; Video scores.	DFMO+CXB/CXB	CXB, 400 mg orally twice a day; DFMO 0.5 g/m^2^/day	6 months	Average percentage change in adenoma polyp counts:CXB + DFMO:− 13.0%; CXB:− 1.0%; average percentage change in adenoma polyp burden:CXB + DFMO:− 40%; CXB:− 27%; video scores:CXB + DFMO:− 0.80; CXB:− 0.33
Jeter 2016 [96]	Sun-damaged skin	Individuals over 40 years of age with visible sun damage to their skin; Individuals with a history of non-melanoma skin cancer received specific cancer treatment at least 30 days prior to enrollment (outside the forearm) or 6 months prior to enrollment (above the forearm)	Mean = 60.4±10.2	44 (28%) /112 (72%)	Average nuclear abnormality (ANA); Proportion of nuclei assigned to the baseline; Histological score	DFMO+Diclofenac/Diclofenac/DFMO	10% DFMO cream (designated 5 cm×5 cm area on the left forearm twice a day); 3% diclofenac gel (designated 5 cm×5 cm area on the left upper arm once a day)	90 days	Average nuclear abnormality (ANA): higher than baseline; Responders with at least a 30% reduction in the nuclear ratio assigned to baseline: DFMO+Diclofenac: 13/45 (29%), Diclofenac: 16/49 (33%), DFMO: 12/50 (24%); histological score: No significant difference from baseline
Bailey 2010 [92]	Non-melanoma skin cancer (NMSC)	Participants had a history of basic or squamous skin cancer, had been treated for basal cell carcinoma or squamous cell carcinoma (stage 0–2), were over 21 years of age, had ECOG status of 0 or 1, and were more than four weeks from prior major surgery or cancer chemotherapy, radiotherapy, or hormone therapy	Mean = 60.9	175 (60%)/116 (40%)	Number of new NMSC events; probability of NMSC occurrence	DFMO/Placebo	DFMO:0.5 g/m^2^/day (0.2 g/ml DFMO liquid or 250 mg DFMO tablet)	3–5 years	Number of new NMSCs: DFMO: 260; placebo: 363; Probability of new NMSCS (/person/year): DFMO: 0.44; placebo: 0.61
Bartels2009 [97]	Actinic skin keratoses	Participants must have at least 2 actinic keratosis on the posterolateral forearm and moderate to severe sunburn as assessed by a dermatologist	Not shown. But there was no difference in the distribution of population characteristics between groups		Percentage of nuclei with ANA>1.0; Percentage of nuclei assigned to the baseline data set	DFMO+triamcilone/DFMO+Eucerin/triamcilone+Eucerin/Eucerin+Eucerin	10% DFMO; 1%Triamcinolone; A dose (1-inch) of the test article or placebo to each posterolateral forearm once daily.	6 months	Changes in percentage of nuclei with ANA>1.0:DFMO+triamcilone:− 6.3%; DFMO+Eucerin:− 2.9%; triamcilone+Eucerin:− 18.7%; Eucerin+Eucerin:4.5%; Changes in percentage of nuclei assigned to the baseline data set:DFMO+triamcilone:− 15.9% vs Eucerin+Eucerin: − 6.3%; DFMO+Eucerin:− 17.9% vs Eucerin+Eucerin:− 10.8%; triamcilone+Eucerin:− 25.6% vs Eucerin+Eucerin:4.8%
Messing 2006 [91]	Low risk superficial bladder cancer	Patients with stage 1 or stage 2 TA or T1 urothelial carcinoma who have undergone complete endoscopic resection or occasional recurrence (less than 1 recurrence per year and no more than 3 total recurrences)	Mean = 64.9	348 (76.7%)/106 (23.3%)	Tumor recurrence	DFMO/Placebo	1 gm daily	12 months (42 months follow-up)	At least 1 case of tumor recurrence:DFMO:103 (46%); Placebo:97 (43%); Progress to TIS or level 3: DFMO:10 (4.4%); Placebo:9(3.9%)
Vlastos 2005 [94]	Cervical intraepithelial neoplasia	Women over 18 years of age who are not pregnant and have biopsy proven squamous intraepithelial neoplasia (CIN 2 or 3, or CIS)	Mean = 31 (18–75)	0/100%	Histopathologic response	Placebo/DFMO 0.125 gm/m^2^/ DFMO 0.5 gm/m^2^	DFMO:0.125 and 0.5 gm/m^2^	28 days	Histopathologic response:0.125 gm/m^2^ DFMO:Complete regression:4; Partial regression:13; No change/progression:30; 0.5 gm/m^2^ DFMO:Complete regression:2; Partial regression:14; No change/progression:31; Placebo:Complete regression:4; Partial regression:13; No change/progression:30

Some researchers have applied DFMO to cancer patients who have regained function after treatment. Recently, a study had demonstrated the evaluation of DFMO as a maintenance treatment regimen for high-risk neuroblastoma. The results showed that the survival status of subjects receiving DFMO for 2 years was maintained well. Therefore, further research on this drug as a maintenance therapy is warranted [91]. It has been evaluated the ability of DFMO to prevent the recurrence of low-risk superficial bladder cancer. The results showed that the tumor recurrence could not be delayed or prevented in low-grade (Grade 1 and 2), superficial (Ta or T1), newly diagnosed or occasionally recurrent bladder urothelial cancer, by using one-year DFMO after the operation of complete resection [92]. 211 participants with a history of non-melanoma skin cancer were randomly assigned to oral DFMO (500 mg/m^2^/day) or placebo for 4–5 years. The primary endpoint was the production of new Non-melanoma skin cancer, with fewer new cancers in the DFMO group than in the placebo group, but there was no statistical difference [93].

Others have used DFMO to treat high-risk groups prone to cancer. For example, a study of familial adenomatous polyposis (FAP) showed moderate synergistic effects of DFMO in combination with celecoxib compared with the use of the non-steroidal anti-inflammatory drug celecoxib alone [94]. In a double-blind randomized trial, DFMO (0.125, 0.5 gm/m^2^) and placebo were used to treat cervical intraepithelial neoplasia (CIN) grade 2–3 patients. There was no significant difference in histopathological responses among the groups [95].

For actinic keratosis (AK), a precancerous lesion that is easy to develop into skin cancer, preclinical studies have shown that the level of skin polyamine is related to the use of DFMO [96], in individuals with signs of actinic keratosis, but the impact of DFMO on the disease is still controversial in clinical trials. It has shown that the combination of DFMO and other drugs can not enhance the activity of treating skin sunburn. The reason may be that the baseline population has mild sun damage [97, 98].

## Conclusions

Over the past decades, polyamine research has continuously progressed. We now have a better understanding of the anabolic pathways and transport processes of polyamines. Moreover, the effects of polyamines on cancer cells have also been explored. This review describes polyamine-related oncogenes and the signaling pathways involved. We found that oncogenes can affect the metabolism and function of polyamines by interfering with the translation and expression of key enzymes. Polyamines can also affect the expression of oncogenes in various ways, thus regulating the physiological function of cancer cells. However, the mechanism of polyamine effect on cancer needs further study, and the types of cancer that have been studied on polyamine metabolism are limited. But the existing research has provided us with new ideas for the treatment of cancer. In fact, some enzyme inhibitors and polyamine analogs have been used as drug interventions in clinical trials and achieved some promising results. This review summarizes the current research on DFMO, a promising drug for cancer chemoprevention. We found that DFMO could slow down the development of cancer in high-risk groups. Nevertheless, DFMO has no significant effect on preventing cancer recurrence for people who have received cancer treatment and recovered their functions, but it is beneficial to maintain their survival status. In summary, we need to further explore the role of polyamines in tumor cells and develop new interventions for cancer treatment and chemoprevention. It is also important to combine drugs targeting the polyamine pathway with other therapies to achieve better outcomes than monotherapies.

## Data Availability

Not applicable.

## Abbreviations

ODC: Ornithine decarboxylase
AdoMetDC: S-adenosine methionine decarboxylase
MTA: 5′-Methyl thio-adenosine
AGMAT: Agmatinase
AZ: Anti enzyme
AZI: Antizyme inhibitor
SSAT: Spermidine/spermine-N-acetyltransferase
PAOX: Polyamine oxidase
SMOX: Spermine oxidase
ROS: Reactive oxides
PTS: Polyamine transport system
GPC1: Glypican 1
DFMO: Difluoromethylornithine
uPAR: Urokinase plasminogen activated receptor
PPARγ: Peroxisome proliferator activated receptor-γ
bFGF: Basic fibroblast growth factor
TGF-β: Transforming growth factor β
PDGF: Platelet-derived growth factor
EGF: Epidermal growth factor
IECs: Intestinal epithelial cells
mTORC1: MTOR complex 1
mTORC2: MTOR complex 2
AP-1: Activator protein 1
eIF-5A: Eukaryotic translation initiation factor 5A
PTCH: Patched
SMO: Smoothed
SUFU: Suppressor of fused
CNBP: Cell nucleic acid-binding protein
FAP: Familial adenomatous polyposis
ER: Estrogen receptor
CIN: Cervical intraepithelial neoplasia
AK: Actinic keratosis
AMPK: Adenosine monophosphate activated protein kinase
